# A Review on Surface-Functionalized Cellulosic Nanostructures as Biocompatible Antibacterial Materials

**DOI:** 10.1007/s40820-020-0408-4

**Published:** 2020-03-14

**Authors:** Mandana Tavakolian, Seid Mahdi Jafari, Theo G. M. van de Ven

**Affiliations:** 1 Department of Chemical Engineering, McGill University, Montreal, QC H3A 0C5 Canada; 2 Pulp and Paper Research Center, McGill University, Montreal, QC H3A 0C7 Canada; 3 Quebec Centre for Advanced Materials (QCAM/CQMF), Montreal, Canada; 4 Department of Food Materials and Process Design Engineering, Gorgan University of Agricultural Science and Natural Resources, Gorgan, Iran; 5 Department of Chemistry, McGill University, Montreal, QC H3A 0B8 Canada

**Keywords:** Cellulose, Nanocellulose, Surface modification, Antibacterial activity, Biocompatibility

## Abstract

As the most abundant biopolymer on the earth, cellulose has recently gained significant attention in the development of antibacterial biomaterials. Biodegradability, renewability, strong mechanical properties, tunable aspect ratio, and low density offer tremendous possibilities for the use of cellulose in various fields. Owing to the high number of reactive groups (i.e., hydroxyl groups) on the cellulose surface, it can be readily functionalized with various functional groups, such as aldehydes, carboxylic acids, and amines, leading to diverse properties. In addition, the ease of surface modification of cellulose expands the range of compounds which can be grafted onto its structure, such as proteins, polymers, metal nanoparticles, and antibiotics. There are many studies in which cellulose nano-/microfibrils and nanocrystals are used as a support for antibacterial agents. However, little is known about the relationship between cellulose chemical surface modification and its antibacterial activity or biocompatibility. In this study, we have summarized various techniques for surface modifications of cellulose nanostructures and its derivatives along with their antibacterial and biocompatibility behavior to develop non-leaching and durable antibacterial materials. Despite the high effectiveness of surface-modified cellulosic antibacterial materials, more studies on their mechanism of action, the relationship between their properties and their effectivity, and more in vivo studies are required.

## Highlights

- The most common chemical treatments of cellulose to synthesize nanostructured cellulose are highlighted.
- Various surface modifications of cellulose to develop non-leaching and durable antibacterial materials are discussed.
- Biocompatibility and antibacterial performance of non-leaching surface-modified cellulosic materials along with their current challenges are discussed.

## Graphical abstract

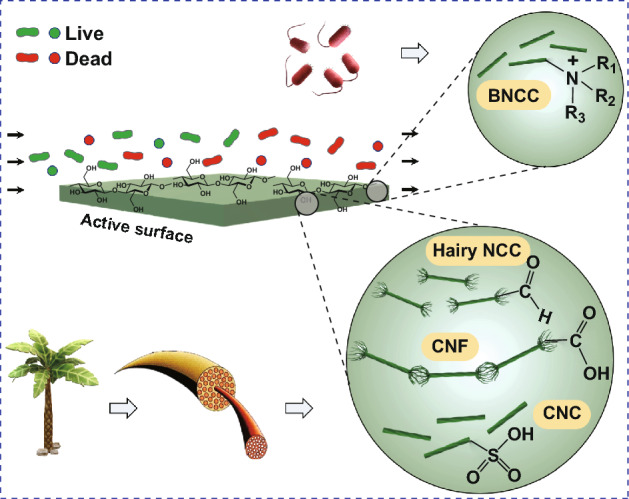

## Introduction

In the past decades, the demand for substituting synthetic materials with naturally derived compounds has increased to protect the environment from undesirable effects. Biopolymers are typically renewable, biocompatible, and biodegradable [1, 2]. Among others, cellulose is the most abundant biopolymer with unique properties and has thus attracted a lot of attention. Apart from the aforementioned properties, it has excellent mechanical properties, low mass density, and is low cost for mass production [3]. In addition, cellulose has high stability against acids, harsh temperatures, and proteolytic enzymes [4, 5]. Cellulose has also been used as the base material to develop hydrogels, films, membranes, aerogels, and capsules for various applications such as food packaging [6–10], water treatment [11, 12], optics and photonics [13, 14], sensing and biosensing [15], and biomedical engineering [16–18].

One of the novel applications of cellulose is the development of cellulosic-based antibacterial materials. In contrast to some other biopolymers with intrinsic biocidal activity (e.g., chitosan [19]), cellulose is not inherently biocidal [20]. For this purpose, it needs to be either chemically modified or grafted with other compounds such as metal nanoparticle (gold, silver, copper, zinc, etc.) [21–23], antibiotics [24, 25], and proteins [26–28]. However, there are some limitations with currently available antibacterial compounds. The emergence of antibiotic-resistant bacteria has limited the use of antibiotics. Also, some metal nanoparticles have been reported to have cytotoxicity effects on mammalian cells [29]. In addition, proteins in free form are very susceptible to environmental conditions and their antibacterial activity diminishes in the long term as a result of conformational changes and agglomeration [20]. Moreover, their covalent attachment to surfaces might reduce their antibacterial activity as some of their functional groups might be affected. Thus, to prevent the loss of antibacterial activity, some antibacterial agents are attached onto carriers non-covalently and their effectivity is achieved through their leaching in the desired area. Leaching of the grafted antibacterial agents is a limitation for applications where long-term activity is desired. This is because as the antibacterial agent is leached out, its concentration in the substrate continually decreases until it falls below its threshold value after which it entirely loses its bactericidal property [30, 31]. In addition, some conventional antibacterial agents (e.g., man-made antibiotics) are associated with environmental problems when they enter municipal wastewater which can be a potential risk to both humans and aquatic organisms [32]. Thus, when the antibacterial agents leach from their carriers through the leaching mechanism, they eventually find their way into various environments such as wastewater which requires further purifications and treatments [33, 34].

Developing biomaterials with a permanent or prolonged antibacterial activity, for which the mechanism of action is necessarily non-leaching, is an alternative strategy to overcome the aforementioned problems. It has been reported that in contact-active antibacterial surfaces, the release of the antibacterial agents is restricted which leads to preserved antibacterial activity for longer periods [35]. In this paper, after a brief overview of cellulose and its different structures, we first describe different methods to modify the surface functional groups of cellulose. Then, antibacterial properties of the produced cellulose derivatives will be discussed. Cellulosic biomaterials with antibacterial activity through grafting with antibiotics, proteins, and metal nanoparticles have been reviewed recently [36]. However, the focus of this review is on developing non-leaching antibacterial cellulosic materials through surface modification based on recent studies.

## An Overview of Cellulose and Its Nanostructures

**Fig. 1:**
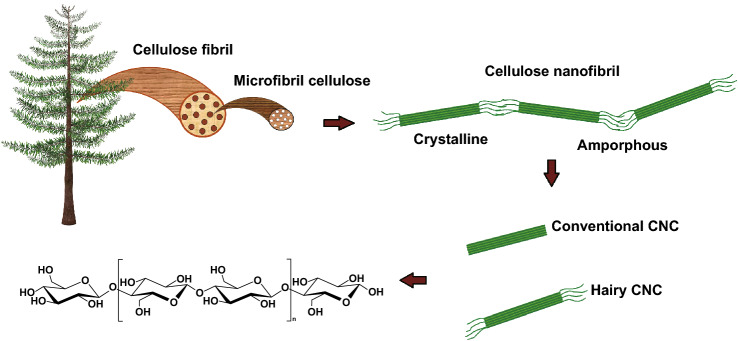
Schematic representation of cellulose structures from resources to molecular level

Cellulose is the most abundant renewable biopolymer on the earth with an annual production of 1.5 × 10^12^ tons [37–39]. It consists of linear glucose rings that are linked by *β*(1 → 4) glycosidic bonds with each ring containing three active hydroxyl groups. Cellulose can be found in various sources including green plant cell wall, some algae, and certain bacteria [40]. Cellulose chains, which are an assembly of several glucose molecules, are bundled together through van der Waals and hydrogen bonds to form three-dimensional networks known as microfibrillated cellulose (MFC). Plant cell wall is made of these microfibrils which are tightly bundled together and difficult to disintegrate due to the strong hydrogen bonding between them [15, 38, 41, 42]. There are different methods to disintegrate MFCs into cellulose nanostructures, one of which is mechanical treatment. However, this process is highly energy-intensive and is not very efficient in terms of disintegration [43]. Chemical treatment is an alternative method which could potentially break the hydrogen bonds between cellulose nanostructures. It could involve functionalizing cellulose fibers with a surface charge, which creates repulsion between the fibers, ultimately leading to their disintegration in the form of either cellulose nanofibers (CNF) or nanocrystalline cellulose (NCC), the latter also known as cellulose nanocrystals (CNC) [44, 45]. Figure 1 shows the hierarchy of cellulose from macroscopic down to the molecular level.

CNCs have been categorized into four different groups depending on the source of cellulose and preparation conditions: CNC, CNF, bacterial nanocellulose (BNCC), and hairy nanocellulose (HNC) [46]. CNCs are rigid nanorods 3–10 nm wide and 100–200 nm long. They can be isolated from cellulose fibrils by acid/enzymatic-assisted hydrolysis to remove their amorphous regions. CNCs are only made of crystalline parts and thus have strong mechanical properties (tensile strength of 7500 MPa and Young’s modulus of 100–140 GPa) due to the high intermolecular bonding [39]. In addition, they have a very high surface area (150–250 m^2^ g^−1^) and a weak thermal expansion coefficient which make them suitable for tissue engineering and cargo carrying [47, 48]. Also, contrary to other common nanostructures such as fullerenes and carbon nanotubes, CNCs are biodegradable [49].

Unlike CNCs, CNFs consist of both crystalline and amorphous regions. CNF is mainly produced by mechanical treatment of a cellulose suspension. It has a dimeter of < 100 nm and a length of several microns [41]. As for BNCC, some types of bacteria such as *Gluconacetobacter xylinus* are able to produce cellulose in their aqueous culture media which are typically used as the source of BNCC. These bacteria produce ribbon-like nanofibrils, and no mechanical process is required to isolate them. BNCC has been shown to have a larger diameter than CNF but have a similar length [50]. Also, compared to CNF, BNCC has a higher crystallinity, purity, and mechanical properties [51, 52].

HNC is a new class of nanocellulose which can be synthesized by oxidizing, solubilizing, and cleaving the amorphous regions of cellulose fibrils. Contrary to conventional CNC, no acid hydrolysis is required to synthesize HNC, leading to a more facile synthesis protocol. Furthermore, compared to conventional CNC which only consists of crystalline regions, HNC nanorods also have amorphous chains (“hairs”) protruding from the both ends, which provide steric stability when suspended. There are different types of HNCs with neutral [53], negative [54], and positive charge content [55] to be chosen for various applications. HNC has the same dimensions as conventional CNC but, when carboxylated, has a higher charge content due to the presence of charged amorphous regions. The functional groups on the amorphous regions at its two ends are more accessible compared to the crystalline parts [56, 57] which makes HNC more reactive. Consequently, HNC is an ideal candidate for surface modification, allowing it to be used as a carrier for various compounds such as drugs, proteins, antibacterial agents, and bioactive ingredients [3, 58, 59].

## Chemical Surface Modification of Cellulose

**Fig. 2:**
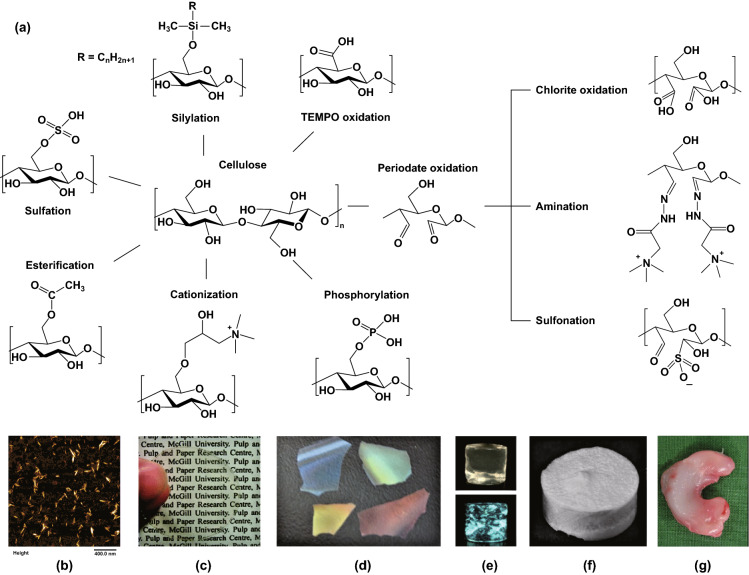
**a** Chemical modifications of cellulose fibers to develop various materials including: **b** hairy nanocellulose as an antibacterial carrier [20], **c** transparent film [59], **d** chiral nematic films [60], **e** hydrogels [61], **f** aerogels [62], and **g** meniscus implant [63]. (Images **b**–**g** are used with permission.)

The high number of hydroxyl groups on the surface of cellulose fibers provides a platform to modify it with different functional groups in order to obtain different properties. Some of the most common functional groups available for functionalization of cellulose are sulfate, carboxyl, aldehyde, phosphate, amino, and thiol groups (see Fig. 2). Surface modification can be used to achieve several goals such as: (i) disintegrating cellulose into its nanostructures to produce CNCs or CNFs, (ii) tuning the surface charge density of cellulose for electrostatic interaction with other molecules, (iii) functionalizing it with functional groups so that it can be covalently grafted with other compounds, and (iv) developing cellulose derivatives with certain properties such as intrinsic antibacterial activity. In this section, we discuss the chemical modifications of cellulose that are mostly being referred to in the current review.

### Aldehyde-Modified Cellulose Derivatives

**Fig. 3:**
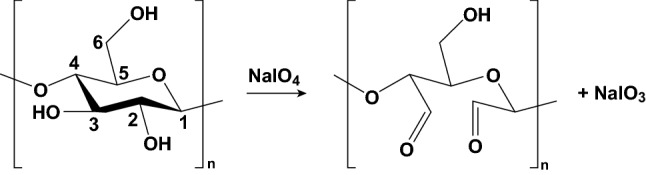
Schematic representation of cellulose reaction with sodium periodate to produce dialdehyde cellulose (DAC)

The reaction of cellulose with periodate leads to cleavage of C_2_ and C_3_ bonds in *β*-*d*-glucose monomer units of cellulose, oxidizing their hydroxyl groups, and formation of 2, 3-dialdehyde cellulose (DAC) [64]. Figure 3 shows the schematic of this reaction with sodium periodate. Full oxidation of cellulose results in conversion of 2 out of 3 hydroxyl groups in each glucose unit. This conversion can be quantified by calculating the degree of substitution (DS) as a measure of the average number of hydroxyl groups which have been converted into aldehydes. Therefore, full oxidation of cellulose corresponds to a DS of 2. DAC with a DS of 2 is completely soluble in water at elevated temperatures (80 °C). On the other hand, partial oxidation of cellulose leads to the formation of dialdehyde modified cellulose fibers (DAMC) [65]. When DS ≈ 1, heating at 80 °C for about 6 h leads to the formation of dialdehyde nanocrystalline cellulose, also referred to as sterically stabilized nanocrystalline cellulose (SNCC). Note that SNCC belongs to the family of HNC. SNCC is a copolymer of DAC and cellulose which is 4–8 nm wide and 100–200 nm long [53]. The aldehyde groups in aldehyde-modified cellulosic compounds (DAC or SNCC) can be readily further converted to carboxylic groups [59, 66], primary alcohols [67], and imines (by a Schiff base reaction) [20] under certain conditions. Thus, DAC is an intermediate to make cellulosic materials for different applications such as dye and heavy metal removal adsorbents [68–70], drug carriers [20, 71], stabilizers for proteins [72, 73], carriers for antibodies [74], and tissue engineering scaffolds [75].

### Carboxyl-Modified Cellulose Derivatives

There are two common methods to develop carboxyl-modified cellulose: (i) TEMPO-mediated^1^ and (ii) chlorite oxidation techniques. However, there are other methods as well such as, hydrogen peroxide oxidation [76].

*TEMPO-mediated* oxidation of cellulose has created an efficient platform to catalyze the conversion of C_6_ hydroxymethyl group of cellulose into carboxyl groups under moderate conditions. The reaction of cellulose with a catalytic amount of TEMPO, NaBr and NaClO as oxidant (4.84 mmol/g cellulose) at pH of 10–11 develops carboxyl groups on the surface of cellulose microfibrils typically with an amount of 0.7 mmol/g cellulose. Further increasing the carboxylic content converts the cellulose fibers to individual CNFs using a mild disintegration for 2–10 min in water with a blender. It should be mentioned that the carboxyl groups are negatively charged and therefore, disintegration into nanostructures is facilitated by the repulsion between the negative charges of carboxylic groups [77–79]. CNFs produced by this method have a width of 3–20 nm and are a few microns long, depending on the source of cellulose. TEMPO-mediated oxidation has also been used to introduce negative charges on the surface of acid-hydrolyzed CNC. The extent of carboxymethylation can be controlled by the amount of primary oxidizing agents (i.e, NaOCl) used in the reaction. As a result of TEMPO-mediated oxidation, initially lots of aldehyde groups are formed and depending on the extent of the reaction, the aldehyde groups can be partially or fully converted to carboxylic groups [80, 81]. It should be noted that excessive TEMPO-oxidation leads to a decrease in the crystal size of produced CNFs because of cellulose delamination [82]. It has also been reported that only half of the hydroxymethyl groups are accessible for this reaction and the rest are buried within the crystalline parts [83].

*Periodate-chlorite* oxidation of cellulose is another common method to make carboxyl-modified cellulose derivatives. After synthesizing DAC by periodate oxidation, the product can be further oxidized by sodium chlorite to convert the aldehyde groups into carboxylic acids and produce 2,3- dicarboxylic cellulose. It has been revealed that a high degree of oxidation decreases the crystallinity of cellulose [64]. On the other hand, partial oxidation results in synthesizing carboxyl-modified cellulose with different crystallinity indices. A study by Yang et al. [54] has shown that partial oxidation of around 50% leads to the formation of three different compounds: (i) CNFs with a length of 0.6–1.8 µm and a width of 120 nm, (ii) HNC with 91% crystallinity index, a length of 120–200 nm and a width of 13 nm, and finally (iii) water-soluble dicarboxylated cellulose. The hairy nanocrystalline cellulose produced by this method is called electrostatically stabilized nanocrystalline cellulose (ENCC) which has a high colloidal stability due to its exceptional carboxylic content (up to ~ 6 mmol g^−1^) as well as the amorphous hairs protruding from the ends where most of the charges are located. The zeta potential of ENCC ranges from − 40 mV at high and low pH values to − 100 mV at physiological pH [84].

### Amine-Modified Cellulose Derivatives

There are several methods to synthesize positively charged CNCs, one of which is the reaction of H_2_SO_4_-hydrolyzed CNCs with epoxypropyl trimethylammonium chloride (EPTMAC) to introduce quaternary ammonium groups onto CNCs. This type of CNC is electrostatically stabilized in aqueous suspensions. This method, although preserving the crystallinity index and morphology of the CNCs, slightly decreases their charge density compared to the initial state [85]. Most of the methods to produce positively charged CNCs are based on post-treatment of H_2_SO_4_-hydrolyzed CNCs [86–88]. The downside of these methods is that they all require extensive acid purification prior to cationization. Yang et al. [55] reported an alternative method that does not require pre-treatment of CNCs with H_2_SO_4_. In this method, after periodate oxidation of cellulose, DAC can further react with (2-hydrazinyl-2-oxoethyl)-trimethylazanium chloride (Girard’s reagent T (GT)) through a Schiff base reaction to form imine bonds and to introduce quaternary ammonium groups on DAC which results in the formation of cationic DAC. Heating the CNFs at 60 °C for 30 min yields positively charged CNCs. In another study for synthesizing positively charged CNCs, cationic DAC was heated for 2 h followed by high intensity sonication to separate the unfibrillated fibers and production of positively charged CNCs with a high yield. It has been shown that the charge content of this product is much higher than the theoretical maximum charge content of traditional CNCs which confirms the presence of hairs.

### Sulfate-Modified Cellulose Derivatives

Sulfation of cellulose can be done during sulfuric acid-catalyzed hydrolysis by esterification of its hydroxyl groups. The most common recipe to synthesize acid-hydrolyzed CNCs is mixing 64 wt% sulfuric acid with cellulose in a ratio of 8.75–17.5 mL g ^−1^at 45 °C for 25–45 min [89]. Sulfuric acid cleaves and dissolves the amorphous chains of cellulose fibers, freeing up the crystalline parts as CNCs [90, 91]. The CNCs produced by this method are negatively charged and form a stable colloidal suspension, although their thermal stability is lower compared to that of cellulose [92]. There is no control over the charge content of CNCs produced by this way, and only slight modification is possible by changing the source of cellulose pulp or significantly changing the reaction conditions, namely acid concentration, temperature, and reaction time [93, 94]. Furthermore, working with high-concentration acids is not desirable from a safety perspective, which further limits the use of this method. Sulfonated CNF can be synthesized without the use of acid hydrolysis and through a green process. This method involves periodate oxidation of cellulose to develop DAC followed by sulfonation using bisulfite. It has been shown that periodate oxidation and sulfonation do not compromise the crystallinity of the produced CNFs, although the developed CNFs have a low charge content (0.18–0.51 mmol g^−1^) [95].

## Antibacterial Activity of Surface-Functionalized Cellulose Nanostructures

Recently, chemical modification of cellulose to obtain antibacterial materials with non-leaching property and prolonged biocidal activity has gained lots of attention [96, 97]. In the following sections, a summary of the most recent studies on modification of cellulose to develop long-lasting non-leaching antibacterial CNCs and CNFs is presented.

### Cationization of Cellulose Nanostructures

Cationic cellulose compounds have intrinsic antibacterial properties. The cell walls of both Gram-positive (G +) and Gram-negative (G −) bacteria have a net negative charge, due to the presence of phosphate groups in the peptidoglycan and phospholipids of the outer membranes of G + and G − bacteria, respectively. Thus, it is desirable to develop materials that provide adsorption ability and high binding affinity for bacterial cells. Cationic CNCs or CNFs can potentially interact with the bacterial cell membrane and compromise its integrity which lead to the leakage of cytoplasmic content and eventually cell lysis [98]. What follows is the discussion of different modifications which lead to the formation of cationic cellulose derivatives with antibacterial activity.

#### Quaternary Ammonium Compounds (QACs)

Among others, quaternary ammonium moieties-bearing molecules are the most studied compounds for developing contact-active surfaces. These materials have been used as antibacterial agents since early 1900s [99]. Their wide structural variety, simple preparation, wide-spectrum of antibacterial activity for both planktonic micro-organism and those found in biofilms, high stability, and good cell membrane penetration properties have made them ideal candidates for developing contact-active surfaces [30, 100]. QACs are composed of a nitrogen atom with a valence of five, attached to four compounds (R_1_-R_4_) including alkyls and heterocyclic radicals as well as small anions such as chloride, bromide, and iodide. The antibacterial activity of QACs mostly depends on their molecular structure and the length of their alkyl chain [101]. For QACs to have antibacterial activity, at least one of the R compounds should be an alkyl with a chain length of C_4_–C_18_. The reason is that high alkyl length is associated with high hydrophobicity which makes the compound compatible with the lipid bilayer of the bacterial cytoplasmic membrane, which is also hydrophobic [30]. It is worth mentioning that alkyl groups have an optimum length after which the accessibility of the QACs for bacteria decreases because of decreased wettability [102]. Consequently, it is crucial to find the optimum alkyl length, which could vary depending on the application of interest, to obtain a high antibacterial efficacy. In general, an alkyl length of C_8_–C_12_ has been reported to have the highest antibacterial activity for two of the most studied bacteria, namely *E. coli* and *S. aureus* [102–104]. In addition to the alkyl length, molecular weight also has a bell-like shape effect on the bactericidal activity of QACs for which the optimal value differs for different compounds [105].

The mechanism of action for QACs is through electrostatic interaction with the negatively charged phospholipids on the bacterial cell membrane. Ion exchange of QACs with Ca^2+^ and Mg^2+^ on the cytoplasmic membrane of bacteria destabilizes bacterial intercellular matrices. Subsequently, QACs diffuse through the bacterial cell wall using their long lipophilic alkyl chain, bind to the bacterial membrane, compromise the permeability of the cytoplasmic membrane, and eventually leading to bacterial death [30, 101]. The release of potassium ions could be used to confirm cytoplasmic membrane disruption, which can be performed using a potassium leakage assay [106]. Antibacterial activity of various QACs on cellulosic supports have already been investigated including 3-(trimethoxysilyl)-propyl- dimethyloctadecyl ammonium chloride [107], quaternized poly(2-(dimethylamino ethyl) methacrylate) (PDMAEMA), cetyltrimethylammonium bromide [108], and 3-chloro-2-hydroxypropyl-tri-methyl ammonium chloride [109].

Table 1 shows some of the most recent studies on cellulose modification with QACs.

**Table 1:** Summary of cellulose derivatives obtained with QACs

QAC type	Cellulosic material	Antibacterial activity	References
Cetyltrimethylammonium bromide	Sulfate-modified CNCs hyperbranched polyethylene ionomers-modified CNCs	NA	[108] [110]
3-chloro-2-hydroxypropyl-trimethyl ammonium chloride	Cellulosic triacetate reverse osmosis membranes	*E. coli* and *S. aureus*	[109]
3-(trimethoxysilyl)-propyldimethyloctadecyl ammonium chloride	Cellulose fibers	*E. coli* and *S. aureus*	[107]
Quaternized 2-(dimethylamino) ethyl methacrylate	TEMPO-mediated CNC	*E. coli* and *S. aureus*	[102]
2,3-epoxypropyl trimethylammonium chloride	Enzymatic microfibrillated cellulose CNFs	*B. subtilis* and *S. aureus* NA	[35] [111]
Trimethoxysilylpropyl octadecyldimethyl ammonium chloride	Cellulose fibers	*B. cereus, E. coli,* *P. aeruginosa*	[112]
(3-Carboxypropyl) trimethylammonium chloride	Cellulose-based photosensitizer	*E. coli* and *S. aureus*	[113]
Girard’s reagent T	Foams made of CNFs	*E. coli*	[114]
Dilinoleic acid-athylenediamine compound	Bacterial cellulose membrane	*S. aureus* and *S. epidermidis*	[115]
*N*-(2-ethoxy-2-oxoethyl)-*N*,*N*-dimethylprop-2yn-1-aminium bromide	Azide-modified cellulose	*E. coli*	[116]
Hexadecyltrimethylammonium bromide	Sulfate-modified CNC	NA	[117]
Poly(isopropanol dimethylammonium) chloride	Filter paper	*E. coli*	[118]
*N*-[(2-hydroxy-3-trimethylammonium) propyl] chitosan chloride	Cellulose acetate electrospun nanofibrous mats	*E. coli* and *S. aureus*	[119]

Pre-treatment of microfibrillar cellulose (MFC) with 2,3- epoxypropyl trimethylammonium chloride (EPTMAC) through the addition of alkali-activated hydroxyl groups of cellulose to the epoxy moiety of EPTMAC leads to cationization of cellulose with different DS values. At DS = 0.18, the cationic MFC showed 100% bactericidal efficacy against *B. subtilis* and a fairly acceptable activity against *S. aureus* (3-log reduction in bacterial population). However, although the procedure was optimized to obtain a high DS and thus a high positive charge content, the charge content was not high enough to effectively inhibit the growth of G- bacteria [35].

To enhance the antibacterial activity of QACs, it has been suggested to use cationic polymers with quaternary ammonium groups instead of monomeric cations. Since polymeric cationic antibacterial compounds have a higher local density of active groups, they can more easily interact with or bind to the negatively charged bacterial cell wall which enhances the disruption of the bacterial cell wall followed by its death [120]. In a study as an example, 2-(dimethylamino)ethyl methacrylate (DMAEMA) was polymerized via a reversible addition-fragmentation chain transfer (RAFT) polymerization from a cellulosic raft agent. Afterward, the tertiary amino groups of the grafted PDMAEMA chains were quaternized using alkyl bromides with different chain lengths (C8–C16). It was shown that, unlike monomeric QAC, cellulose-g-PDMAEMA was effective against G- bacteria (i.e., *E. coli*). The length of alkyl chain and the degree of quaternization are two parameters affecting the antibacterial activity. The cellulose-g-PDMAEMA with the maximum degree of quaternization (34%) and an alkyl chain of 8 carbons (C_8_) showed the highest activity against *E. coli* [30].

Direct covalent bonding of QACs onto cellulose without the need of any linker was performed using a dual-functional QAC which makes the total process more economical. 3-(trimethoxysilyl)-propyl- dimethyloctadecyl ammonium chloride has an antibacterial quaternary ammonium site and a reactive silane site (SiH_4_) which can react with the –OH groups in cellulosic compounds, resulting in the formation of strong Si–O–Si bonds. The antibacterial cellulosic surface developed using this method shows a reasonable stability in a wide range of temperatures (25–90 °C) and pH values (1.5–10). It is notable, however, that at temperatures beyond 90 °C and pH values less than 1.5 or above 10 the Si–O bonds begin to hydrolyze which decreases the stability of these compounds. That said, these contact-active antibacterial surfaces showed a prolonged efficacy after repeated usages. In terms of antibacterial effectiveness, 1 h treatment of *P. aeruginosa*, *B. cereus*, and *E. coli* with quaternized cellulose surfaces showed a significant biocidal activity with complete inactivation for *E. coli* and *P. aeruginosa* and tenfold reduction in CFU counts of *B. cereus* [112].

**Fig. 4:**
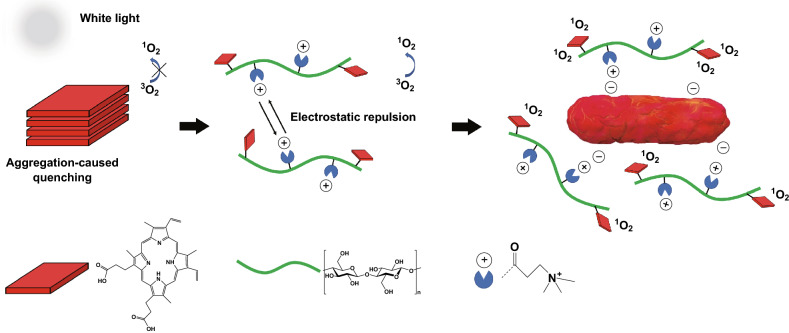
Antibacterial mechanism for cellulose-based photosensitizers under white-light irradiation [113]

QACs not only have intrinsic antibacterial activity, but also show synergistic effects when used with other antibacterial agents. One example is their usage with porphyrin-based photosensitizers, which are used in photodynamic therapy as an alternative to antibiotics due to the emergence of antibiotic-resistant bacteria. Photoporphyrins have been shown to be able to effectively kill antibiotic-resistant bacterial strains via generation of reactive oxygen species (ROS) using light irradiation. ROS radicals are potent oxidants which compromise the integrity of bacterial cells and since they cause nonspecific damage, the chance of resistance build-up in bacteria is very low [121, 122]. Although photoporphyrins are less prone to form resistance in bacteria, they have some limitations such as their hydrophobicity which makes them insoluble in aqueous media. Furthermore, they can easily aggregate due to their planar molecular structure which hampers their efficacy [123]. Moreover, these compounds are electrically neutral and cannot effectively interact with the negatively charged bacterial membrane. As a result, most of the generated ROS get inactivated during a long-distance diffusion [124, 125]. QACs can be used to resolve these issues as well as to synergistically enhance their antibacterial activity. Utilizing the hydroxyl groups of cellulose, QACs and photoporphyrin can be covalently attached onto cellulose via an esterification reaction to make dual-functional compounds. The positive charge of QACs deals with the water-insolubility and weak interaction of photoporphyrins with the bacterial membrane. Also, the electrostatic repulsion between QAC groups inhibits the aggregation of porphyrins. It has been shown that at relatively low concentrations of photosensitizers and a low dosage of white-light irradiation (2.4 J cm^−2^), QAC-photoporphyrin modified cellulose can effectively kill antibiotic-resistant *E. coli* and *S. aureus* strains. Notably, the mechanism of action for this complex is through ROS generation and interaction of QACs with the bacterial cell membrane (see Fig. 4) [113].

Another example of the synergistic effects of using QACs is in water sanitation. QACs have been used to develop antibacterial filters for water purification with an extremely high efficiency of 7 orders of magnitude reduction in live bacteria with a simple filtration process. For this purpose, filter paper is first coated with a cationic polyelectrolyte binder (CPE) with poly(isopropanol dimethylammonium) chloride (PIDMAC) as the CPE binder. Then, amphiphilic block copolymer micelles loaded with a hydrophobic biocide, triclosan, is attached to the fibers of filter paper via CPEs with polystyrene-block-polyacrylic acid (PS-b-PAA) as the block copolymer. It has been shown that triclosan and PIDMAC have a synergistic effect that is responsible for the extremely high observed antibacterial activity. Specifically, PIDMAC and triclosan alone reduce *E. coli* viability by 4 and 1 orders of magnitude, respectively. However, their combination shows a substantial synergy with a reduction of 7 orders of magnitude in bacterial viability [118].

#### Aminoalkyl/Aminosilane

**Fig. 5:**
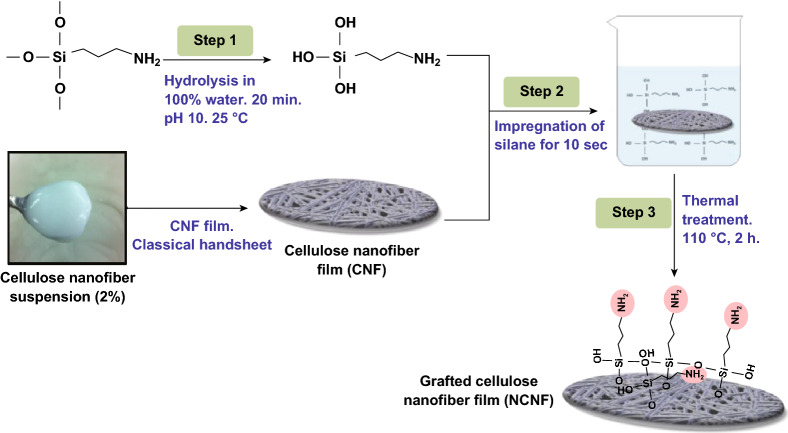
Schematic illustration of grafting (3-aminopropyl) trimethoxysilane onto cellulose nanofibers in water. Adapted from [134] with permission

APMS ((3-aminopropyl)trimethoxysilane) is one of the most studied aminoalkyls (otherwise known as aminosilane) grafted with cellulose derivatives for various applications [126–130]. Inspired by the intrinsic antibacterial activity of chitosan due to the presence of free amino groups on its backbone [19, 131], APMS is used to graft aminoalkyl on the surface of cellulose derivatives. The chemical grafting is started by an intermediate silanol group formation and then continues with the reaction of silanol to hydroxyl groups on the substrate. The silanol groups can also be subjected to self-condensation, resulting in polysiloxane formation. Saini et al. have studied various parameters including solvent type, initial concentration of silane, pH, etc., to minimize self-condensation and maximize grafting efficiency [132, 133]. The grafting of APMS and CNFs involves three steps which are shown schematically in Fig. 5. Briefly, these steps are hydrolysis of silane coupling agents and their conversion into silanol groups in water, adsorption of silanol groups onto CNFs, and finally covalent bonding between silanol groups and hydroxyl groups of CNF and Si–O–C formation. The high specific surface area of the adsorbent (CNFs) and complete miscibility of the adsorbate (silane) in the solvent improve the grafting efficiency. Consequently, choosing a proper solvent in which the adsorbate is highly soluble is crucial. Also, increasing the initial concentration of the adsorbate is another way to improve the grafting efficiency [134].

**Fig. 6:**
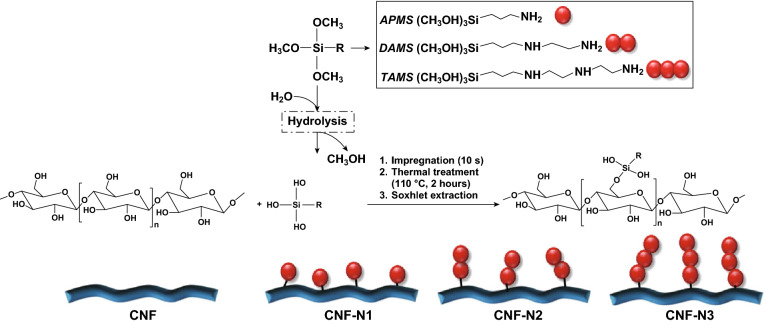
Schematic illustration of the aminosilane grafting on the surface of CNF films. Adapted from [137] with permission

APMS has been used to graft aminoalkyl groups on the surface of bacterial CNFs using a silane chemical grafting approach [97]. Aminoalkylated bacterial cellulose membranes have shown instant lethal activity against *S. aureus* and *E. coli* while being non-toxic to human adipose-derived mesenchymal stem cells. The antibacterial activity of APMS-modified bacterial CNF, apart from its polycationic nature, is due to the length of alkyl chains. Increasing the alkyl chain length results in increased lipophilicity, which affects their interaction with the cytoplasmic membrane of bacteria [104, 135]. It has been shown that increasing the alkyl length up to 10 carbons yields amino-alkyl modified bacterial cellulose with a broad spectrum of antibacterial and antifungal activity as well as biocompatibility with human embryonic kidney 293 cells (HEK293) [136]. In addition to the alkyl chain length, increasing the number of amino groups is another strategy to enhance the antibacterial activity of these compounds. Three different aminoalkyls: APMS, 2-aminoethyl 3-aminopropyl trimethoxysilane (DAMS), and 3-2-(2-aminoethylamino) ethylamino propyl-trimethoxysilane (TAMS) have been studied by grafting them onto hydroxyl groups of the CNFs (see Fig. 6). TAMS with the highest aminoalkyl chain showed the highest grafting efficiency as well as the highest stability and antibacterial activity against G + bacteria [137].

#### Quaternized Halamine

*N*-halamines have been used extensively as biocidal compounds since 1980s. *N*-halamines consist of one or more halogen atoms that are linked to the nitrogen-containing compounds, which control the stability and release rate of free active halogens into the environment [98]. They have been grafted to different materials including cellulose, chitosan, nylon, and polyurethane to develop antibacterial surfaces [138–140]. The mechanism of action of the *N*-halamines is through direct contact of oxidative halogens (i.e., Cl^−^ or Br^−^) with thiol or amino groups of proteins which leads to inactivation of bacterial cells or inhibition of their growth [141]. Owing to their long-term stability in aqueous and dry storage conditions, regenerability, non-toxicity, low cost, and highly efficient broad-spectrum activity against bacteria, viruses, fungi, and yeasts, *N*-halamines have found various applications in water treatment, food packaging, latex paint, household sanitations, and air cleaning materials [142–146]. Cellulose is a suitable candidate to be functionalized with *N*-halamines due to abundance of hydroxyl groups on its backbone which can be easily grafted with *N*-halamine [147]. Various *N*-halamine precursors with reactive agents including epoxy groups and organosiloxane have been grafted onto cellulose, and durable antibacterial compounds have been developed.

One of the unique characteristics which distinguishes *N*-halamines from other bactericidal agents is regenerability. Once the bacteria are killed upon exposure to *N*-halamines, they can be regenerated by exposure to a diluted household bleach solution [148]. Nevertheless, *N*-halamines are poorly soluble in water, which limits their application. Quaternized *N*-halamine can be used to resolve this issue. In addition to the enhanced water solubility of quaternized *N*-halamines, they have a notable antibacterial activity due to the synergistic effect between quaternary ammonium and *N*-halamine. One example of such compounds is s-triazine-based quaternized *N*-halamine, which can be prepared at low temperatures and non-acidic conditions. This compound can be synthesized by substituting two reactive chlorine groups of cyanuric chloride with 4-amino-2,2,6,6-tetramethylpiperidine and *N*-(2- aminoethyl) pyridinium chloride. In addition, it readily reacts with cellulose without the need for any linkers. The modified cellulose produced this way was shown to be able to induce a 6-log reduction against *S. aureus* and *E. coli* after only 1–5 min exposure. Furthermore, almost 50% of the oxidant chlorine in *N*-halamine molecules was retained after 50 cycles of washing and 30 days of storage, while the rest 50% could be recovered upon exposure to household diluted bleach solution [147].

### Aldehyde-Modified Cellulose Nanostructures

**Fig. 7:**
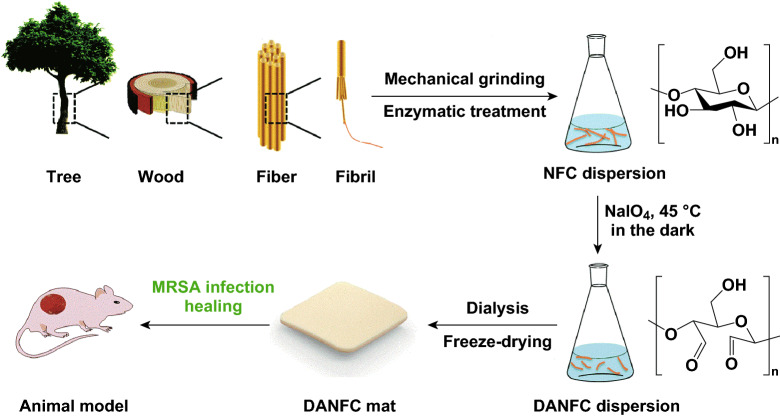
Schematic representation of DANFC production for wound healing applications. Adapted from [151] with permission

Oxidized regenerated cellulose (ORC) has shown antibacterial activity against a wide range of bacteria and has been widely used in hospitals for wound dressing applications. The mechanism of action for ORCs is by inducing an acidic environment [149, 150]. It is hypothesized that 2,3-dialdehyde nanofibrillated cellulose (DANFC) has the same mechanism of action as ORC. The acidic pH of DANFC is due to the presence of dialdehyde groups. DANFC has shown a good antibacterial activity against both *S. aureus* and methicillin-resistant *S. aureus* (MRSA) (see Fig. 7). It was shown that higher aldehyde content is associated with lower pH, and hence a higher bactericidal activity. This characteristic can be exploited for wound dressing applications [151].

**Fig. 8:**
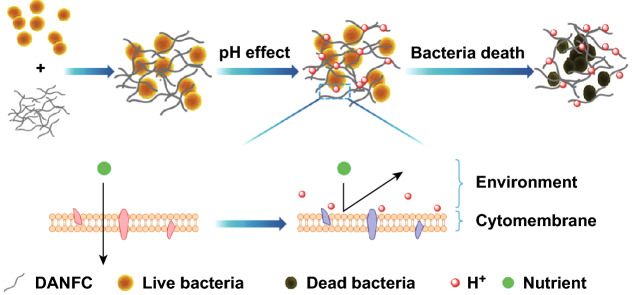
Scheme of the antibacterial mechanism for DANFC. Adapted from [151] with permission

Intact skin has a slightly acidic pH between 4 and 6 due to the secretion of organic acids by keratinocytes to regulate bacterial flora and prevent infection. However, an infected wound has a neutral pH (7–7.5) which is the ideal pH for bacteria to grow [152, 153]. Low pH affects the activity of proteins and hydrolases in the bacterial cytomembrane, affects the permeability of bacterial membranes, and also affects the absorbance of nutrients, as shown in Fig. 8 [151]. Furthermore, low pH is fatal to a broad spectrum of bacteria and although there are some bacteria that can survive in acidic environments [154, 155], it is less likely that they develop resistance to bacteria as antibiotics [149]. DANFC has also shown a decent blood compatibility with a hemolysis < 1%. In addition, it has shown a good thrombogenicity effect with an average thrombin generation lag time of around 20 min, which further indicates its potential for wound dressing applications. The cytotoxicity of DANFC with different aldehyde contents was also measured with human microvascular endothelial cell line and umbilical vein endothelial cells. At concentrations < 1 mg mL^−1^, both NFC and DANFC with different aldehyde contents showed cell viability of around 90%. However, at higher concentrations, the cell viability with respect to DANFC with high aldehyde content (1.5 mmol g^−1^) decreased to around 79% while others remained around 90% [151]. This decrease in cell viability might be due to the acidity of aldehyde groups [156]. Dialdehyde microcrystalline cellulose (DAMC) has also shown an antibacterial activity against both G + and G − bacteria. Similar to DANFC, higher aldehyde content shows better bactericidal activity. However, the reported minimum inhibitory concentration (MIC) for DAMC is relatively high. For instance, the MIC of DAMC against *S. aureus* was 15 mg mL^−1^ when the aldehyde content was around 6.5 mmol g^−1^ [157].

### Carboxyl-Modified Cellulose Nanostructures

Carboxyl-modified CNCs are not potent antibacterial agents but can be used as carriers for antibacterial agents or drugs [36, 46]. In this section the biocompatibility of the carboxyl-modified CNCs is discussed. Surface charge, in addition to size and hydrophobicity, is one of the most crucial parameters that affect the performance of nanocarriers especially their cellular uptake, which is an indicator of biocompatibility [158]. In addition, charge content is associated with colloidal stability since nanoparticles are prone to aggregation in the presence of salt [54]. Aggregation of nanoparticles in blood or plasma extensively decreases their cellular uptake in the body. Conventional CNC, which can be synthesized by acid hydrolysis, has a low charge content (up to 0.8 mmol g^−1^) and has a high tendency to aggregate when subjected into complex fluids such as blood. Thus, it is desirable to develop CNCs with a tunable surface charge to prevent their aggregation in physiological conditions while not affecting their biocompatibility and cellular uptake.

**Fig. 9:**
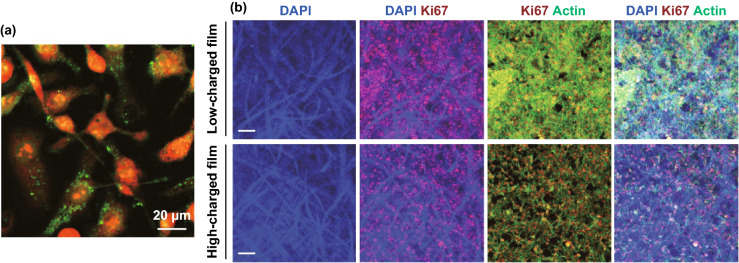
**a** ENCC internalized by HeLa cells. Cells were stained with Eth-1 and Alexa Fluor 633 (both are red) and ENCC conjugated with fluoresceinamine (green dots) [58]. **b** Representative confocal images of the expression of Ki67 (in red) and cytoskeleton marker actin (in green) in Hela cells growing into HC and LC films. Scale bars are 200 µm [163] (Figures are used with permission.). (Color figure online)

Hairy nanocellulose with negative charge, namely ENCC, with its extremely high charge content (up to 6.6 mmol g^−1^) provides a highly colloidal stable platform even when exposed to high salt concentrations or serum-containing media. It has been shown that ENCC is stable at ionic strengths up to 2 M while conventional CNC aggregates at 30 mM. In addition, it offers continuous control over its charge content with carboxylic charge content ranging from 1.7 to 6.6 mmol g^−1^ [54]. The effect of ENCC on cell viability was tested on cells from human colon (Caco-2), kidney (MDCK), cervix (Hela), and macrophage cell lines (J774). MTS assay showed that carboxylic content of below 3.8 mmol g^−1^ has a negligible effect on cell metabolic activity. However, beyond 3.8 mmol g^−1^ carboxylic charge content, a clear charge-dependent loss of metabolic activity of cells was observed. The high charge content of ENCC is beneficial because they can be loaded with desired cargos (fluorophores, drugs, antibacterial agents, DNA strands, etc.), while still retaining enough carboxylic groups to prevent aggregation and decreased cellar uptake. Darkfield hyperspectral imaging and confocal microscopy clearly confirmed the presence of ENCC in the cells. This along with the results of Live/Dead assays, which shows the amount of compromised cells is comparable with control (both contain less that 10% compromised cells), confirms the biocompatibility of the ENCC (see Fig. 9a). It was also observed that higher uptake was achieved with a higher charge density of ENCC [58]. Moreover, it shows that negatively charged nanoparticles, such as ENCC, can penetrate into cell walls and thus cationic charge is not essential.

One of the indicators to assess the biocompatibility of biomaterials is the level of their endotoxins such as bacterially derived lipopolysaccharides (LPS), which is an inducer of inflammatory cytokines in human cells. Unpurified materials containing LPS lead to inflammatory responses and compromise the biocompatibility. Endotoxins can be removed from synthesized materials by heating at high temperatures (> 250 °C) or through exposure to basic and acidic solutions of 0.1 M concentration [159]. Ultrapure CNF was developed based on a modified TEMPO-mediated oxidation method using sodium hydroxide as a pre-treatment. It was shown that the produced CNF by this method contains an endotoxin level of < 50 EU g^−1^ which is lower than the currently commercially available biomaterials (< 100 EU g). Also, a CNF-dispersion at a concentration of 50 µg mL^−1^ showed no cytotoxicity against Normal Human Dermal Fibroblasts and Human Epidermal Keratinocytes. This was confirmed by measuring 27 different cytokines after exposure of the cells to CNF, and no cytokine secretion above the level of the control was observed [160]. In another study on the effect of CNF concentration on cytotoxicity, it was shown that concentrations up to 1 mg mL^−1^ do not induce any cytotoxicity or oxidative stress in the L929 cells, nor any necrosis and apoptosis in human peripheral blood mononuclear cells. However, higher concentrations of CNF were shown to inhibit the proliferation and metabolic activity of the cells [161].

In another study, to further confirm the biodegradability of cellulosic materials, *Cladophora* green algae was used as the source of nanocellulose and was modified with TEMPO to functionalize it with carboxylic groups. It was shown that TEMPO-mediated nanocellulose promotes fibroblast adhesion and provides a high cell viability. The negative charges on nanocellulose favor the protein adsorption on the nanocellulose film which promotes cell adhesion [162]. CNF-based films and hydrogels with different porosity and charge density were prepared as scaffolds to support 3D cell culture for tissue engineering applications. It was shown that films and hydrogels with a lower carboxylic charge content (1.5 mmol g^−1^) can promote survival rate and proliferation of tumor cells (Hela and Jurkat cells) with a cell toxicity < 5% after 72 h of treatment. However, high surface charge density hinders the cell’s biological responses leading to a slower growth rate and higher cytotoxicity (see Fig. 9b) [163]. For further reading on biocompatibility of nanocellulose, one can refer to other relevant studies [164, 165].

### Bacteriophage-Modified Cellulose Nanostructures

Bacteriophage, or simply phage, is a form of virus that can inject its DNA or RNA and replicate inside the bacteria. Because of that, when exposed to an infected surface, bacteriophages can multiply and grow in number rapidly, resulting in the release of a large number of progeny phages which further intensifies their replication [166]. Because of its efficiency and its specificity against harmful bacteria, the phage is a promising potential contender to replace traditional synthetic antibiotics [167, 168]. Bacteriophages are usually negatively charged at their head and thus can bond with positively charged particles through electrostatic interaction [169–173]. In addition, the active groups on the phage such as amino and carboxylic acid groups allow for functionalization through covalent attachment, resulting in a much more durable attachment.

T4 lysozyme, a product of gene *e* of T4 bacteriophage, is one of the common bacteriophages for medical studies. Lysozyme, also known as muramidase, is a common antibacterial enzyme that operates via hydrolyzing the 1,4-*β*-linkage between *N*-acetylmuramic acid and *N*-acetylglucosamine in the peptidoglycan layer of bacteria, which leads to cleavage of the bond, cell lysis, and eventually bacterial death [174]. Lysozyme is mostly effective against G + bacteria due to the absence of peptidoglycan layer in G- bacteria [20]. T4 bacteriophage, which is effective against *E. coli*, produces lysozyme to facilitate the cleavage of the cell wall and release of the virion progeny from the infected bacteria [175]. T4 bacteriophage is effective against both G- and G + bacteria. Its activity against G-bacteria is the result of its amphipathic α-helix interaction with the negatively charged lipopolysaccharides in the bacterial cell membrane which leads to penetration of the phage into the membrane [176]. T4 bacteriophage has been successfully immobilized on CNCs without any genetic or chemical modification. It has been shown that the enzymatic (both lytic and hydrolytic) and antibacterial activity of T4 bacteriophage can be preserved to a great extent through immobilization on surface-modified CNC. In a recent study, CNC was firstly synthesized through a single-step procedure with ammonium persulfate. Afterward, T4 bacteriophages were covalently immobilized on two types of CNC, each containing either only carboxylic groups or amine groups. In the former, carbodiimide (EDC)-activated carboxylate groups of CNC were reacted with the amine groups of T4 bacteriophages through a bioconjugation reaction and formation of amide groups. In the latter, ammonium salt of CNC (CNC-COO-NH_4_) was firstly oxidized with sodium periodate to form dialdehyde CNC. Then, amino-functionalized CNC (Am-CNC) was developed through reaction with ethylenediamine solution at a pH of 10. Some of the amine groups were then activated with glutaraldehyde to react with T4 bacteriophage through a Schiff base reaction. It was shown that both methods gave the same immobilization yield, but glutaraldehyde-modified Am-CNC resulted in a higher activity of T4 bacteriophage. The antibacterial activity of T4-immobilized CNC was tested against four model bacteria, two G- (*E. coli* and *Ps. Mendocina*) and two G + (*M. lysodeikticus* and *Corynebacterium sp.*). T4 bacteriophages that were immobilized on Am-CNC showed higher antibacterial activity (less MIC value) than that in free form, while those immobilized on EDC-activated CNC were found to be less effective. One possible reason could be the higher zeta potential of Am-CNC conjugates compared to that in both free form and EDC-activated CNC conjugates [177]. A higher zeta potential is desirable for antibacterial agents as they can reach negatively charged bacteria and deactivate them more efficiently. It is worth mentioning that the orientation of the bacteriophage when immobilized on the solid support could significantly affect its activity. Different immobilization methods lead to different orientations of the bacteriophages. Thus, covalent attachment might compromise the activity of bacteriophage by covering phage tail fibers, which are used to target bacteria [178]. Consequently, it is crucial to find the right candidates for both the solid support and immobilization method while using bacteriophages. Although bacteriophages are very efficient antibacterial agents, the wrong choice of treatment can compromise their activity substantially.

## Concluding Remarks

Despite the remarkable advances in the development of antibacterial materials, several issues in developing biocompatible and biodegradable surfaces with long-lasting antibacterial activities and relatively low costs remain unresolved which require further investigations and improvements. Cellulose, the most abundant biopolymer on the earth, has a great potential to address these issues. While cellulose in its pristine form does not have intrinsic antibacterial properties, it has an abundance of functional groups which can be exploited in different ways. For instance, they can be directly used to attach natural antibacterial agents to make cellulosic antibacterial compounds. This study reviews those antibacterial cellulosic compounds that entirely owe their antibacterial activity to the added functional groups rather than to the use of external antibacterial agents such as antibiotics, metal nanoparticles, proteins. Such compounds provide long-lasting, and high-efficiency properties with non-leaching antibacterial mechanism. Their exclusive benefit over the latter antibacterial cellulosic materials comes directly from the non-leaching mechanism of action, which eliminates the loss of antibacterial agent. For instance, for the case of antibacterial metal nanoparticles or antibacterial peptides grafted onto cellulosic materials, the leached antibacterial agents tend to aggregate, which leads to lower capture efficiencies and lower effective lifespans. Surface-active antibacterial surfaces resolve these issues by eliminating the leaching step; hence they are more long-lasting. However, their major drawback is their passive mechanism of action, i.e., for them to be functional, the bacteria need to be actively exposed to these surfaces. Despite that, these materials are ideal candidates for applications where such contact is automatic such as antibacterial filters, water purification, food packaging, wound dressing, and antibacterial coatings especially in healthcare environments to prevent cross-infection.

More specifically, in this review, we studied the effect of various functional groups (i.e., quaternary amine, aldehyde, quaternized halamine, etc.) on the antibacterial performance of cellulosic materials. Quaternary ammonium compounds are among most studied functional groups. There are several parameters that affect the efficacy of these materials such as alkyl length, molecular weight, and the extent of functionality. However, there is some discrepancy in the reported studies. For example, some report that by increasing the alkyl length the bactericidal activity increases as the hydrophobicity increases and the compounds become more compatible with the lipid bilayer. While others report that the alkyl length has an optimum value after which the antibacterial activity decreases. The same issue exists for the effect of molecular weight. These issues remain to be further investigated to design cellulosic surface-active compounds with more tunable properties. Finally, while cellulosic materials are widely being used in food packaging, water treatment applications, and for biomedical applications, despite extensive in vitro studies confirming their biocompatibility, further in vivo studies are required to pave the way for clinical trials.

## Footnotes

1 (2,2,6,6-Tetramethylpiperidin-1-yl)oxyl or oxydanyl which is commonly known as TEMPO.
